# The investigation of adrenal involvement in carbapenem resistant Acinetobacter baumannii sepsis: experimental mouse model

**DOI:** 10.3906/sag-2001-163

**Published:** 2021-09-07

**Authors:** Esma ERYILMAZ-EREN, Gökçen DİNÇ, Olgun KONTAŞ, Emine ALP, Mehmet DOĞANAY

**Affiliations:** 1 Department of Infectious Diseases and Clinical Microbiology, Kayseri City, Education and Research Hospital, Kayseri, Turkey; 2 Department of Microbiology and Clinical Microbiology, Faculty of Medicine, Erciyes University, Kayseri, Turkey; 3 Department of Pathology, Faculty of Medicine, Erciyes University, Kayseri, Turkey; 4 Turkey Republic Ministry of Health, Ankara, Turkey; 5 Department of Infectious Diseases and Clinical Microbiology, Faculty of Medicine, Erciyes University, Kayseri, Turkey

**Keywords:** *Acinetobacter baumannii*, adrenal insufficiency, corticosteroids, mice model, sepsis

## Abstract

**Background/aim:**

In the last years, incidence of carbapenem resistant *Acinetobacter baumannii* sepsis is increasing with high mortality. However, it is not clear whether this is due to inadequate antimicrobial choice or a more severe clinical course. We aimed to evaluate the inflammation and adrenal involvement in the carbapenem resistant *A. baumannii* by using experimental mouse model sepsis.

**Materials and methods:**

Balb/c female mice were randomly put into control and three sepsis groups (*A. baumannii* susceptible to carbapenem-CSAB-, *A. baumannii* resistant to carbapenem-CRAB-, *Escherichia coli*). A total of sixty mice were included in this study with each group having 15 mice. Mice were sacrificed 72 h after bacterial inoculation, and blood was taken from each mouse for the assessment of cytokines and corticosterone. Both adrenal glands were dissected; one was used for culture and the other was used for histopathological examination. Bacterial loads of organs were calculated as CFU/g. The histopathological changes, bacterial levels in adrenal and cytokine and corticosterone levels were assessed and compared among the groups.

**Results:**

The bacterial level was higher in *E. coli* (108, 45 ±30, 55 log10 CFU/g) (mean±SD) than other sepsis groups. The lowest level of corticosterone was observed in the *E. coli* group (p < 0.001). TNF alpha level was highest in the CRAB and *E. coli* group and this difference was statistically significant than control group (p < 0.05). The IL-6 level in CRAB was significantly higher than the control group (10, 20 pg/mL). The adrenal gland congestion was significantly severe in all the sepsis groups compared to the control. In the group comparison, congestion was significantly more severe in the *E. coli* group than in CSAB and CRAB groups.

**Conclusion:**

Adrenal involvement and inflammatory reactions are seen in *E. coli* sepsis and in CRAB sepsis. These findings will be focused on in future clinical trials.

## 1. Introduction

Sepsis is defined as a life-threatening organ dysfunction caused by dysregulated host response to infection [1]. It is an inflammatory disease that can cause impairment of tissue perfusion, multiple organ failure, shock, and even death. Sepsis is defined by the World Health Organization (WHO) as a global health problem, and the reported mortality rate of sepsis in hospitalized patients ranges from 30 to 45%. Despite recent advances in treatment and technology, the sepsis mortality rate is still relatively high. Although sepsis pathogenesis is not clearly understood, it is known that antigens and toxins of microorganisms induce inflammation. Many systems and cells are activated by bacterial products such as antigens, toxins, enzymes, etc. and lead to pathophysiological events such as the release of cytokines, neutrophil migration through chemotaxis, impairment of endothelium permeability, hyperactivation of coagulation pathway. These events can result in perfusion impairment, hypoxia, edema, and necrosis. These had to organ dysfunction and/or organ failure that consists of the central nervous system, lung, kidney, liver, and/or hemopoietic systems [2, 3].

In cases of increased stress in the body such as trauma, pain or inflammation, the hypothalamo-hypophyseal adrenal axis (HPA) is activated. The level of glucocorticoid increases with the release of adrenocorticotropic hormone (ACTH) stimulus [4, 5]. It is known that the adrenal glands are affected by certain infections such as meningococcemia, brucellosis, tularemia, etc. HPA is affected by the direct invasion of microorganisms into the organs or due to toxin effect during sepsis. Adrenal failure seems to be one of the most important prognostic factors in the outcome of sepsis. Adrenal insufficiency leads to inadequate stress response and may increase mortality. Currently, adrenal insufficiency is still a debatable issue [6].

According to the recent treatment guidelines, hydrocortisone treatment is recommended (200 mg / day) for patient with septic shock, who do not respond adequately to fluid replacement and vasopressor treatment [1]. However, the effect of steroid treatment on reducing mortality in sepsis is still controversial [3]. In previous clinical study with sepsis, basal cortisol level was higher in the patients with shock than without shock. Basal cortisol level was also lower in the patients with gram-negative bacterial sepsis than in the patients with gram-positive bacterial sepsis [7]. In recent years, the incidence of nosocomial sepsis has been increased due to multidrug resistant (MDR) bacteria [8]. One of the most common gram-negative bacteria is MDR acinetobacter baumannii in the intensive care unit (ICU). According to Xx University’s Hospital Infection Control Surveillance report (2016), A. baumannii is the most common cause of ICU-acquired infections [9]. It is also known that MDR A. baumannii has a higher mortality than antibiotic susceptible A. baumannii. However, it is not clear whether or not this is due to an inadequate choice of antimicrobials or if there is a more severe clinical reason [10, 11]. The aim of this study is to evaluate whether or not the inflammation and adrenal involvement in the carbapenem resistant A. baumannii differs from carbapenem susceptible gram-negative bacteria in experimental mouse model sepsis.

## 2. Materials and methods

This experiment was carried out at the Experimental and Clinical Research Center at xx. The study was approved by the local ethics committee for animal experimentation.

### 2.1. Animal

Balb/c female mice aged 20–24 weeks weighing 40–50 g were used in the study. All mice were caged in groups of three or four and given ad libitum access to food and water. Animals were acclimatized in a 12-h light/dark cycle.

### 2.2. Bacteria

The study was performed with strains of carbapenem susceptible A. baumannii (CSAB) ATCC 17978, carbapenem resistant A. baumannii (OXA-51, OXA-58, PER-1 positive; CRAB) and *E. coli* ATCC 25922. CRAB was a clinical isolate obtained from the blood culture of a bacteremic patient at the hospital

### 2.3. In vivo study

The mice were randomly put into two main groups: control group and sepsis group. The sepsis groups were also divided into three subgroups: CSAB, CRAB, and *E. coli* study group. A total of sixty mice were included in this study. The control group and sepsis subgroups each consisted of 15 mice. Sepsis was developed by an intraperitoneal injection (ip) of 0.1 mL bacterial suspensions, which had been determined for each group in the preliminary study (Table 1). All mice were sacrificed at 72 h with an overdose of anesthetic (100 mg/kg ketamine hydrochloride, Pfizer, Turkey) and 10 mg/kg xylazin (Xylazin bio %2, 20 mg/mL flakon, Bioveta). Intracardiac blood samples were taken for cytokine analysis and corticosterone. Tissue samples were taken from the lung, liver, heart, and right adrenal gland and cultured on tryptic soy agar (Merck, Germany) to check the development of sepsis. Sepsis was defined with observation of the bacterial growth at least two organs excluding peritoneum. Quantitative culture was performed, and results were given as CFU per gram for adrenal gland [10,11]. Also, 10-fold dilutions of right adrenal glands homogenates were processed to obtain quantitative counts as CFU/g would be used to compare the control and study groups. The left adrenal glands were used for histopathological examination.

### 2.4. Cytokine and corticosterone analysis

Serum samples were stored at −20 °C for corticosterone, TNF-alpha and IL-6 according to the kit procedure instructions using enzyme-linked immunosorbent assay (ELISA) method (FineTest, Mouse CORT (Corticosterone) ELISA Kit EU3108, Mouse TNF-alpha (Tumor Necrosis Factor Alpha) ELISA Kit EM0183, Mouse IL-6 (Interleukin 6) ELISA Kit EM0121) The cytokine levels were measured by ELISA plate reader (GloMax Microplate Reader, PROMEGA).

### 2.5. Histopathological examination

The left adrenal glands from all the mice were fixed in 10% neutral buffered formalin solution. After 24 h of fixation, all tissues were blocked in paraffin and 5 micrometer sections. They were prepared and stained with hematoxylin eosin following by being examined under light microscope. In the microscopic examination, three layers of the cortex and the adrenal glands medulla were evaluated separately. All layers of the adrenal gland were evaluated for congestion, neutrophil infiltration and other possible changes. The changes were recorded semiquantitatively as 0: none, 1: mild, 2: moderate, 3: severe to allow statistical comparisons (Table 2). Pictures are given as an example of the evaluation in Figure.

### 2.6. Statistical analysis

Before the study, a power analysis was studied. The results of the power analysis showed that 15 mice per group were required to achieve 80% power. Bacterial counts from quantitative cultures of adrenal gland tissue, serum corticosterone, IL-6 and TNF-alpha levels and tissue histological grades according to the groups were compared and analyzed using SPSS 22.0 (SPSS Inc., Chicago, IL, USA). Results of the microbiological study were recorded as log10. Shapiro–Wilk test for normality distribution analysis was performed before analysis. A one-way ANOVA was used to test for differences among the groups. A post-hoc Tukey test was performed to determine which group had a different result. Data were presented as mean ± standard deviation. A p-value of < 0.05 was considered significant statistically.

## 3. Results

This study involved 60 mice, but 2 mice were excluded from the CRAB group due to death within 24 h (Table 1).

### 3.1. Microbiological examination

Sepsis developed in all study groups. Bacterial growth in organ cultures is shown in Table 3. The bacterial levels of adrenal glands were higher in *E. coli* than CRAB and CSAB groups. The differences were proven to be statistically significant (p < 0.001).

### 3.2. Histopathological examination

In the microscopic examination, normal adrenal tissue was observed in the control group (Figure A). Neutrophil leukocyte infiltration was not observed in the sepsis groups. However, congestion of the adrenal gland was detected in the groups (Figure B). The adrenal gland congestion was significantly more severe in the sepsis groups compared to the control group (Table 3). In the comparison of the sepsis groups, congestion was significantly more severe in the *E. coli* than in CSAB and CRAB groups (p < 0.05).

The histopathological examination, vacuolated cells between the deep parts of the cortex and the medulla were observed in some adrenal glands (Figure C and D). These cells were also graded and recorded. Vacuolated cells were observed in 6 mice in the CSAB group, 12 mice in the CRAB group, and 14 mice in the *E. coli* group; however, vacuolated cells were not present in the control group. There were more vacuolated cells in the CRAB and *E. coli* group than in the control and CSAB groups. Statistical analysis showed that the difference between the control group of two sepsis groups (CRAB and *E. coli* groups) was significant (p < 0.001).

### 3.3. Hormone and cytokine assays

The corticosterone levels were lower in the *E. coli* group than any other sepsis groups and the control group. Also, corticosterone level was lower in the CRAB than CSAB (Table 3). The difference was found to be statistically significant (p < 0.05).

The highest TNF-alpha mean level was in the CRAB group (Table 3). The mean of TNF-alpha level was significantly higher in CRAB and *E. coli* than the control group (p < 0.05). However, this difference was not statistically significant.

CRAB group had the highest IL-6 level (Table 3). In statistical analysis only, IL-6 mean level in the CRAB group was statistically significantly higher than the control group (p = 0.009).

## 4. Discussion

Throughout the world, severe sepsis and septic shock still remain major health problem. Despite improvements in medical care, severe sepsis is still associated with high mortality [12]. Rapid activation of the adrenal glands glucocorticoid and catecholamine production is a fundamental component of the stress response and is essential for survival of the host. Multiorgans including adrenal glands are also affected by sepsis leading to organ dysfunctions. In these patients, the plasma levels of ACTH and cortisol are often affected. The HPA axis and glucocorticoid actions are shown to be impaired in many critically ill patients. In previous studies, the adrenal insufficiency rate has been reported to vary between 10 to 20% and may be as high as 60% in cases with septic shock [13]. It is not known which bacterial agent has more influence on the adrenal glands. However, certain agents such as Neisseria meningitis, Mycobacterium tuberculosis are known to affect adrenal glands [14].

Many different microorganisms are responsible for adrenal involvement in sepsis. In bacterial infections, many pathological conditions such as massive bleeding, abscess, and granulomas are observed. Hemorrhage is the most common lesion in adrenal. There is also a correlation between the presence of bacterial involvement in the adrenal gland and adrenal hemorrhage [15]. The current study, histopathological examination of the adrenal glands revealed no leukocytes, hemorrhages and necrosis in any of the layers. However, congestion was observed in different layers among the groups. Profuse congestion was observed in the *E. coli* and CRAB groups, whereas less severe congestion was observed in CSAB group. The difference in the effect on the adrenal glands and the severity of sepsis between mice infected with CSAB or CRAB strain cannot be associated with carbapenem resistance occurs due to the difference in the virulence of the strains.

It has been reported in the literature that vacuolated cells in corticomedullary complements of the adrenal glands are present in mice and disappear with puberty reputation. These cells are not seen in human adrenals^1^. The presence of these cells in mouse studies is accepted as normal. The current study showed that the vacuolated cells increased in the severe sepsis groups. This may suggest the effect of stress on adrenal glands. More studies are needed to study the effect of stress on adrenal glands as well as. Hormonal levels should be studied from these vacuolated cells.

Marik et al. found that 25% of patients in 59 sepsis cases in their study had adrenal insufficiency and 17% had hypothalamo-hypophyseal axillary failure [16]. In another study that consisted of in 189 severe sepsis patients, adrenal insufficiency was observed in 10% of patients. Adrenal insufficiency is correlated with increasing mortality [17]. The corticosterone level was significantly lower in *E. coli* group. In addition, corticosterone levels in the CRAB group were lower than in the CSAB group. This suggests that adrenal insufficiency develops in the sepsis of E. *coli* and CRAB.

Adrenal functions are related to inflammation. Endotoxemia increases circulating IL-1, IL-6 and TNF-a, and these cytokines acutely and transiently activate HPA, thus, increase the release of cortisol [18]. On the other hand, products such as TNF-α and corticostatin can inhibit the production of adrenal function and cortisol [19, 20]. In particular, inflammatory mediators have been shown to have suppressive effects on adrenal function. Prolonged exposure to cytokines may result in an altered response of the hypothalamic-pituitary axis. Similarly, the chronic increase in IL-6 may lead to a reduction in ACTH production; it has been shown that TNF-a may cause a reduction in corticotrophin releasing hormone (CRH) stimulation and ACTH production in the adrenal function [21, 22]. Low ACTH levels have been observed in patients that were diagnosed with severe sepsis or systemic inflammatory response syndrome [18]. Patients with severe sepsis were evaluated and found to have lower mean cortisol levels in the group with higher mortality in a study [22]. Obviously the HPA axis relation with sepsis is not clearly defined. In the inflammatory response, there is some evidence that cortisol production increases in patients, but nearly half of the patients with septic shock have an inadequate response to metyrapone synthesis which suggests a reduction in cortisol synthesis [23]. In our study, IL-6 and TNF-α levels were high in the *E. coli* and CRAB group where the cortisol level was measured at the lowest level in the *E. coli* group. The bacterial level in the adrenal gland was significantly higher in the *E. coli* group, whereas the level of corticosterone was lower in *E. coli* and CRAB (Table 3).

Several studies on septic shock have reported that IL-6 and TNF-α levels are elevated and have a correlation between cytokines levels cytokines and mortality [18]. In another study, high TNF-α levels were reported as a high predictive value for mortality for gram-negative sepsis [24]. In the presence of inflammation, IL-6 has an enhancing effect on serum corticotropin and cortisol levels [19]. Andaluz-Ojeda et al., in a sepsis study with 17 immunomodulators, found that IL-6 levels were correlated with mortality rates between 3 and 28 days. Wu et al. found that there were higher levels of IL-6 in septic shock patients when compared to nonshock sepsis patients and group with higher mortality [25, 26]. In our study, TNF alpha levels were significantly higher in the *E. coli* and CRAB groups compared to the control group, and the highest TNF alpha level was detected in the CRAB group. IL-6 levels were higher in three of the sepsis groups than in the control group. Statistically, only the CRAB group was statistically significantly different from the control group.

In this experimental mouse model sepsis, corticosterone levels were lower in the CRAB group than CSAB group. Also, cytokine levels (TNF alpha and IL-6) were highest in the CRAB sepsis than CSAB sepsis. In conclusion, a similarity adrenal involvement and inflammatory reactions are seen in *E. coli* sepsis and in CRAB sepsis, which have high virulence.

In this study, 15 mice in each group were determined by power analysis. However, it is an important limitation that two mice in the CRAB group were not included in the study because they died of sepsis. Further studies with larger working groups are needed. The authors believe that these findings will shed light on future clinical trials.

## Figures and Tables

**Figure:**
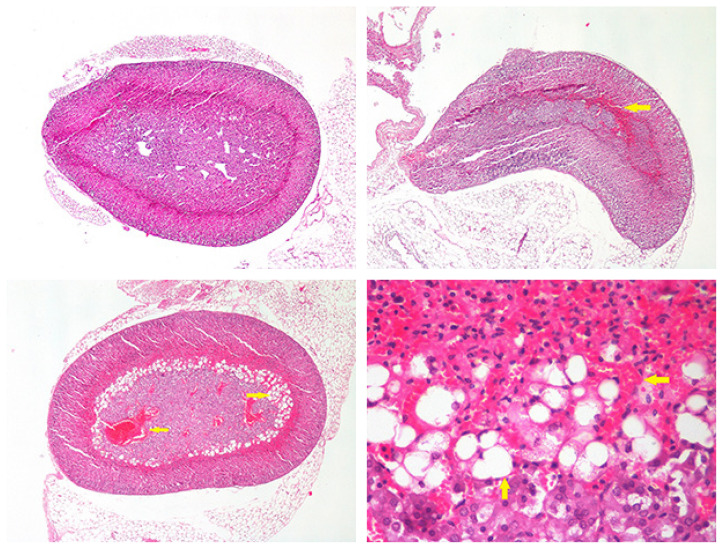
(HE: Hematoxylin eosin) A: Normal adrenal tissue (HEx40); B: Septic adrenal tissue; Mild congestion (HEx40); C: Septic adrenal tissue; severe congestion and fatty cell accumulation and in medulla, (HEx40); D: Septic Adrenal Tissue severe congestion and fatty cell accumulation and in medulla (HEx100)

**Table 1:** Study groups for the experimentation

Group	n	Inoculum dose	72 h
**Control Group**			
Group 1	**15**	Serum Physiologic	Sacrification
**Sepsis Groups**			
Group 2 CSAB	**15**	4x10^8^ cfu/mL (0.1 ml)	Sacrification
Group 3 CRAB	**13** *	3x10^8^ cfu/mL (0.1 ml)	Sacrification
Group 4 *E. coli*	**15**	1x10^8^ cfu/mL (0.1 ml)	Sacrification

* Two mice were excluded from the study died within 24 has they.

CSAB: Carbapenem susceptible *A. baumannii*.

CRAB: Carbapenem resistant *A. baumannii*.

**Table 2:** Scores of histopathological examination

	None	Mild	Moderate	Severe
Congestion	0	1	2	3
Neutrophil infiltration	0	1	2	3
Vacuolated cells	0	1	2	3

**Table 3:** Bacterial load and congestion in adrenal tissue, corticosterone and cytokines levels in the groups

PARAMETERS	GROUPS				
	Control (n=15)	CSAB (n=15)	CRAB (n=13)	*E. coli* (n=15)	*P*
Bacterial count, log10 CFU/g Mean ±SD	NG	71,46±26,12	55,67±31,86	108,45 ±30,55^a^	< 0.001
Corticosterone Mean ±SD (μg/mL)	76,66±3,31	80,63±4,22	71,96±4,87^b^	62,71±9,96^c^	< 0.001
TNF alpha Mean ±SD (pg/mL)	195,84±46,48	217,07±33,04	267,07±31,55^d^	250,62±73,20^e^	< 0.001
IL-6 Mean ±SD(pg/mL)	7,24±2,62	8,77±2,41	10,20±2,36^f^	9,52±1,98	0.010
Congestion Mean ±SD	0,46±0,63	4,30±1,70^g^	3,73±2,08^h^	6,00±1,64^i^	0.009
Bacterial growth in liver	NG	10/15	8/13	7/15^j^	0.001
Bacterial growth in lung	NG	12/15	9/13^k^	14/15^l^	< 0.001
Bacterial growth in heart	NG	10/15	11/13	11/15^m^	< 0.001

NG: No Growth

CSAB: Carbapenem Susceptible A. baumannii

CRAB: Carbapenem Resistant A. baumannii

a p=0.001 with CSAB, p<0.001 with CRAB and control

b p=0.003 with CSAB,

c p=0.001 with CRAB, p<0.001 with CSAB and control

d p<0.001 with control,

e p=0.004 with control

f p=0.009 with control

g p<0.001 with control,

h p<0.001 with control

i p=0.036 with CRAB, p=0.002 with CSAB, p<0.001 with control

j p<0.001

k p=0.030,

l p<0.001

m p<0.001

## Footnotes

1 U.S. Department of Health and Human Services, National toxicology program (2015). Nonneoplastic atlas, Adrenal gland. Available from: Population Fact Sheets: Turkey [online]. Website https://ntp.niehs.nih.gov/nnl/endocrine/adrenal/index.htm [2015].

## References

[1] Singer M Deutschman CS Seymour CW Shankar-Hari M Annane D The Third International Consensus Definitions for Sepsis and Septic Shock (Sepsis-3) JAMA 2016 315 801 810 10.1001/jama.2016.0287 26903338 PMC4968574

[2] Angus DC van der Poll T Severe Sepsis and Septic Shock New England Journal of Medicine 2013 369 840 851 10.1056/NEJMra1208623 23984731

[3] Venkatesh B Finfer S Cohen J Rajbhandari D Arabi Y Adjunctive Glucocorticoid Therapy in Patients with Septic Shock New England Journal of Medicine 2018 378 9 797 808 10.1056/NEJMoa1705835 29347874

[4] Chrousos GP The hypothalamic-pituitary-adrenal axis and immune-mediated inflammation New England Journal of Medicine 1995 332 20 1351 1362 10.1056/NEJM199505183322008 7715646

[5] Annane D The Role of ACTH and Corticosteroids for Sepsis and Septic Shock: An Update Frontier of Endocrinology 2016 20 7 70 10.3389/fendo.2016.00070 PMC4913096 27379022

[6] Guarner J Paddock CD Bartlett J Zaki SR Adrenal gland hemorrhage in patients with fatal bacterial infections Modern Pathology 2008 21 9 1113 1120 10.1038/modpathol.2008.98 18500257

[7] Aygen B Inan M Doğanay M Keleştimur F Adrenal functions in patients with sepsis Experimental and Clinical Endocrinology and Diabetes 1997 105 3 182 186 10.1055/s-0029-1211749 9228516

[8] Peleg AY Seifert H Paterson DL *Acinetobacter baumannii*: emergence of a successful pathogen Clinical Microbiology Reviews 2008 21 538 582 10.1128/CMR.00058-07 18625687 PMC2493088

[9] 2016 Surveying Records Infection Control Board, Erciyes University

[10] Dinc G Demiraslan H Elmali F Ahmed SS Alp E Antimicrobial efficacy of doripenem and its combinations with sulbactam, amikacin, colistin, tigecycline in experimental sepsis of carbapenem-resistant *Acinetobacter baumannii* New Microbiologica 2015 38 1 67 73 25742149

[11] Dinc G Demiraslan H Elmali F Ahmed SS Metan G Efficacy of sulbactam and its combination with imipenem, colistin and tigecycline in an experimental model of carbapenem-resistant *Acinetobacter baumannii* sepsis Chemotherapy 2013 59 5 325 329 10.1159/000356755 24525528

[12] Angus DC Wax RS Epidemiology of sepsis: an update Critical Care Medicine 2001 29 109 116 10.1097/00003246-200107001-00035 11445744

[13] Kanczkowski W Sue M Zacharowski K Reincke M Bornstein SR The role of adrenal gland microenvironment in the HPA axis function and dysfunction during sepsis Molecular and Cellular Endocrinology 2015 408 241 248 10.1016/j.mce.2014.12.019 25543020

[14] Paolo WF Jr1 Nosanchuk JD Adrenal infections International Journal of Infectious Disease 2006 10 343 353 10.1016/j.ijid.2005.08.001 PMC7110804 16483815

[15] Tormos LM Schandl CA The significance of adrenal hemorrhage: undiagnosed Waterhouse-Friderichsen syndrome, a case series Journal of Forensic Sciences 2013 58 4 1071 1074 10.1111/1556-4029.12099 23458363

[16] Marik PE Zaloga GP Adrenal insufficiency during septic shock Critical Care Medicine 2003 31 141 145 10.1097/00003246-200301000-00022 12545007

[17] Annane D Sebille V Toche G Raphael JC Gajdos P A 3-level prognostic classification in septic shock based on cortisol levels and cortisol response to corticotropin JAMA 2000 283 1038 1045 10.1001/jama.283.8.1038 10697064

[18] Soni A Pepper GM Wyrwinski PM Ramirez NE Simon R Adrenal insufficiency occurring during septic shock: incidence, outcome, and relationship to peripheral cytokine levels The American Journal of Medicine 1995 98 3 266 271 10.1016/S0002-9343(99)80373-8 7872343

[19] Jäättelä M Ilvesmäki V Voutilainen R Stenman UH Saksela E Tumor necrosis factor as a potent inhibitor of adrenocorticotropin-induced cortisol production and steroidogenic P450 enzyme gene expression in cultured human fetal adrenal cells Endocrinology 1991 128 623 629 10.1210/endo-128-1-623 1702707

[20] Zhu Q Solomon S Isolation and mode of action of rabbit corticostatic (antiadrenocorticotropin) peptides Endocrinology 1992 13M31 1413 1423 10.1210/endo.130.3.1311240 1311240

[21] Mastorakos G Chrousos GP Weber JS Recombinant interleukin-6 activates the hypothalamic-pituitary-adrenal axis in humans Journal of Clinical Endocrinology and Metabolism 1993 77 1690 1694 10.1210/jcem.77.6.8263159 8263159

[22] Marik PE Zaloga GP Adrenal insufficiency in the critically ill: a new look at an old problem Chest 2002 122 1784 1796 10.1378/chest.122.5.1784 12426284

[23] Annane D Pastores SM Arlt W Balk RA Beishuizen A Critical illness-related corticosteroid insufficiency (CIRCI): a narrative review from a Multispecialty Task Force of the Society of Critical Care Medicine (SCCM) and the European Society of Intensive Care Medicine (ESICM) Intensive Care Med 2017 43 12 1781 1792 10.1007/s00134-017-4914-x 28940017

[24] Surbatovic M Popovic N Vojvodic D Milosevic I Acimovic G Cytokine profile in severe Gram-positive and Gram-negative abdominal sepsis Scientific Reports 2015 16 5 11355 10.1038/srep11355 PMC4468818 26079127

[25] Andaluz-Ojeda D1 Bobillo F Iglesias V Almansa R Rico L A combined score of pro- and anti-inflammatory interleukins improves mortality prediction in severe sepsis Cytokine 2012 57 3 332 336 10.1016/j.cyto.2011.12.002 22197776

[26] Wu HP Chen CK Chung K Tseng JC Hua CC Serial cytokine levels in patients with severe sepsis Inflammation research 2009 58 7 385 393 10.1007/s00011-009-0003-0 19262987

